# Gene-based multiple trait analysis for exome sequencing data

**DOI:** 10.1186/1753-6561-5-S9-S75

**Published:** 2011-11-29

**Authors:** Jingyuan Zhao, Anbupalam Thalamuthu

**Affiliations:** 1 Human Genetics, Genome Institute of Singapore, 60 Biopolis Street 02-01, Singapore 138672

## Abstract

The common genetic variants identified through genome-wide association studies explain only a small proportion of the genetic risk for complex diseases. The advancement of next-generation sequencing technologies has enabled the detection of rare variants that are expected to contribute significantly to the missing heritability. Some genetic association studies provide multiple correlated traits for analysis. Multiple trait analysis has the potential to improve the power to detect pleiotropic genetic variants that influence multiple traits. We propose a gene-level association test for multiple traits that accounts for correlation among the traits. Gene- or region-level testing for association involves both common and rare variants. Statistical tests for common variants may have limited power for individual rare variants because of their low frequency and multiple testing issues. To address these concerns, we use the weighted-sum pooling method to test the joint association of multiple rare and common variants within a gene. The proposed method is applied to the Genetic Association Workshop 17 (GAW17) simulated mini-exome data to analyze multiple traits. Because of the nature of the GAW17 simulation model, increased power was not observed for multiple-trait analysis compared to single-trait analysis. However, multiple-trait analysis did not result in a substantial loss of power because of the testing of multiple traits. We conclude that this method would be useful for identifying pleiotropic genes.

## Background

The common disease/common variant hypothesis states that common variants contribute substantially to common diseases [1,2]. Following this hypothesis, genome-wide association studies have successfully detected associations with common variants. However, such common variants explain only a small proportion of the phenotypic variation. Many of the as yet undetected common variants may have small effect sizes; therefore they are not expected to contribute significantly to the missing heritability. An alternative theory, the common disease/rare variant hypothesis, argues that a large number of rare variations with moderate to high penetrances account for genetic susceptibility to common disease [1]. Recently, deep-resequencing studies of candidate genes have provided some evidence supporting the common disease/rare variant hypothesis [3]. Although various statistical methods have been developed to detect associations with common variants for common diseases, these methods are inefficient for rare variants because of the small number of observations for each single rare variant. One feasible method for rare variant analysis is to pool multiple rare variants within a gene or region and to test their joint effect. This category of methods has been reviewed by Dering et al. [4].

Some genetic association studies examine a qualitative trait, such as the case-control status and some additional correlated quantitative traits. For example, a genetic study of diabetes may examine the diabetic status and other related phenotypes, such as body mass index and other lipid profiles. Similarly, a glaucoma study may explore the related endophenotypes, such as central corneal thickness, intraocular pressure, and maximum vertical cup-to-disc ratio. One way to analyze these data is to perform single-trait analyses separately. An alternative way is to perform a multiple-trait analysis, which potentially has improved power to identify the pleiotropic variants for these traits [5,6].

Univariate test statistics or *p*-values of multiple traits may not be independent because of the environmental or genetic correlations among multiple traits. Hence some classical methods of combining independent *p*-values, such as Fisher’s method, are not directly applicable to the analysis of multiple correlated traits. Our purpose in this paper is to develop a test statistic for combining these correlated univariate statistics by considering the correlation structure among multiple traits. Motivated by a recently proposed approach [7], we developed a gene association test to test the joint effect of multiple variants within a gene on multiple correlated traits. The proposed method considers genes as basic units and uses the weighted sum [8] to combine the effects of multiple variants. The test statistic of multiple traits is the linear or quadratic combination of the univariate test statistics. It is likely that some rare variants may contribute to only a subset of available traits. Therefore we also conduct an alternative test on a preselected subset of multiple traits.

## Methods

Let *Y* = (*Y*_1_, …, *Y_m_*)*^T^* denote the *m* available traits. Assume that the gene *k* has *L* genotyped single-nucleotide polymorphisms (SNPs), including both common and rare ones. In the first step, the genetic score *S_j_* of the gene *k* for an individual *j* is calculated using the weighted sum of all SNPs within the gene. Second, a univariate test is performed to establish the association of genetic scores with all the traits separately. Then, a gene-level association test using the linear or quadratic combination of single-trait univariate statistics is constructed for multiple traits. Finally, the optimal subset of traits is selected for multiple-trait analysis. The details of the various steps are described in what follows.

### Gene score using weighted sum

The weighted-sum gene score assigns different weights to each variant based on the estimated allele frequencies [8]. The score for gene *k* for individual *j* is given by:(1)

where *I_ij_* is the number of minor alleles for SNP *i* in individual *j*,(2)(3)

and *m_i_* is the total number of minor alleles for SNP *i* in all *n* individuals. In the original study [8], the allele frequencies were estimated only for the control subjects. Because multiple-trait analysis needs to analyze multiple quantitative traits as well as the disease status, in the present study we estimate the allele frequencies using all individuals.

### Association test for multiple traits

Let *S* = *S*_1_, *S*_2_, …, *S_n_*)*^T^* denote the scores for gene *k* for *n* individuals. The test statistic for testing the association of *S* with each trait *Y_l_* (*l* = 1, …, *m*) is denoted *Z_l_*, and it is assumed to asymptotically follow *N*(0, 1) under the null hypothesis. The choice for *Z_l_* is discussed in the Results section. The test statistic for the combined multiple traits (CMT) method is a quadratic or linear combination of *m* univariate test statistics *Z*_1_, …, *Z_m_*.

The CMT method is motivated by a recently proposed approach [7] to test the joint effect of multiple correlated SNPs. Because the test statistics *Z*_1_, …, *Z_m_* are assumed to asymptotically follow *N*(0, 1), the joint distribution of the random vector **Z** = (*Z*_1_, …, *Z_m_*)*^T^* is asymptotically multivariate normal *N_m_*(0, **Σ**), where **Σ** is the correlation matrix of **Z**. The quadratic combination statistic  is given by:(4)

and it is distributed asymptotically as a chi-square distribution with *m* degrees of freedom if **Σ** is full rank [9]. Because **Σ** is unknown, it is estimated by permutations. The phenotypes are permuted, and the *m* univariate test statistics are computed under each permutation. The estimation of **Σ** is given by:(5)

under *N* such permutations. The *p*-value of the quadratic statistic () can be approximated by the chi-square quantile.

An alternative statistic for multiple correlated traits is the linear combination statistic :(6)

which is distributed asymptotically as *N*(0, 1) [9]. Here also the covariance matrix **Σ** is replaced by its estimate , obtained from permutations. The *p*-value of the linear statistic can be approximated by the standard normal quantile. Note that the linear combination statistic  may result in a loss of power if the direction of association is not the same for all of the traits. By contrast, if the effects on these traits are in the same direction, it is possible that the power of  may be better than the power of . In general,  is more robust than .

### Association test for the optimal subset of multiple traits

It is likely that some of the relevant rare variants are associated with only a subset of traits. In this case, combining all the test statistics using the CMT method may result in a loss of power because of the high degree of freedom in the chi-square distribution. Therefore we propose an association test for optimally combined multiple traits (OCMT) using a preselected subset of these traits. To select the optimal subset, we use the CMT method to calculate the *p*-values for all possible subsets with at least two traits. The subset with the minimum *p*-value is selected as the optimal one, denoted A*. For any subset with at least two traits *A*, the quadratic combination statistic  is given by:(7)

where **Z***_A_* and **Σ***_A_* are the subvector and submatrix of **Z** and **Σ**, respectively. The *p*-value  is obtained from the chi-square quantile. The *p*-value of A* () is given by:(8)

To control type I error, the *p*-value of the OCMT method is obtained using a permutation procedure that is based on the permutation of phenotypes. For each permutation *π*, the subset with the minimum *p*-value is selected as the optimal subset, denoted A*(*π*). The *p*-value of OCMT () is defined as the proportion of permutations with , where  is the *p*-value of A*(*π*).

## Results

We applied the CMT and OCMT methods to the GAW17 simulated mini-exome data sets [10]. The results are reported in two parts. In the first part, the power of the proposed method is compared to the power of the single-trait analysis. Initially, the CMT and OCMT methods were proposed and applied to the GAW17 data set without the knowledge of the simulation model. These original results were presented at the GAW17 meeting. Because the simulation model used to create the GAW17 data was discussed at the workshop, we reran the analysis with the knowledge of the simulation model. Here we present the results of the revised analysis based on the knowledge of the simulation model. In the second part, we present some insights into the false-positive rate of the CMT method.

For each gene, we tested each trait separately using the *t*-test statistic of the *β* coefficient corresponding to the gene score in the logistic regression (for the disease status D) or the linear regression (for quantitative traits Q1, Q2, and Q4) adjusted for three covariates (Age, Sex, and Smoking status). The test statistic *Z_l_* (*l* = 1, …, *m*) was obtained from the inverse normal distribution transformation of the *t*-test statistic [7] and was assumed to have a standard normal distribution.

The association tests for multiple traits were performed by using the CMT method with the quadratic combining statistic. The *p*-value was approximated using the theoretical quantiles of the chi-square distribution. The correlation matrices among the test statistics *Z*_1_, …, *Z_m_* were estimated by 1,000 permutations. In addition, the association test using the OCMT method was performed, and its *p*-value was obtained from another set of 1,000 permutations. Given a predefined significance level *α*, the power of any association test was defined as the proportion of 200 replicates that returned a *p*-value less than or equal to *α*.

The samples in the GAW17 data set were collected from six cohorts, but population stratification was not considered in the simulation model. We performed the association tests with and without corrections for stratification. To correct for stratification, we corrected genotypes and phenotypes using the first 10 principal component scores derived using Eigenstrat [11].

### Power of single- and multiple-trait analyses

Table 1 presents the power to detect the Q1 causal genes at a significance level of 0.05. Without adjusting for stratification, the univariate test for Q1 has a power greater than or equal to 0.3 for eight genes. With the adjustment, only four genes (*FLT1*, *KDR*, *VEGFA*, and *VEGFC*) still have a power greater than or equal to 0.3. For Q2 and Q4, most of the genes have a power less than 0.1. Some of the genes also are associated with the disease status (power ≥ 0.3).

**Table 1:** Power to detect the causal genes on Q1 at the 0.05 significance level

Gene	Q1	Q2	Q4	D	Q1+D	Q1+Q2 +Q4+D	OCMT	Subset with the highest power (power)
*ARNT*	0.185 0.145	0.050 0.050	0.055 0.040	0.070 0.065	0.180 0.165	0.140 0.115	0.160 0.135	Q1+D (0.180) Q1+D (0.165)
*ELAVL4*	0.520 0.045	0.050 0.035	0.060 0.045	0.030 0.045	0.465 0.040	0.390 0.040	0.380 0.045	Q1+D (0.465) Q1+Q4 (0.055)
*FLT1*	1.000 0.995	0.075 0.075	0.055 0.040	0.700 0.475	1.000 0.995	1.000 0.985	1.000 0.980	Q1+D (1.000) Q1+Q2 (1.000)
*FLT4*	0.865 0.030	0.055 0.040	0.060 0.060	0.290 0.085	0.765 0.065	0.600 0.045	0.655 0.045	Q1+D (0.765) Q2+D (0.085)
*HIF1A*	0.565 0.025	0.220 0.080	0.040 0.020	0.280 0.095	0.530 0.055	0.450 0.060	0.465 0.065	Q1+D (0.530) Q2+D (0.090)
*HIF3A*	0.030 0.090	0.055 0.040	0.065 0.060	0.030 0.050	0.010 0.080	0.035 0.065	0.045 0.060	Q2+Q4 (0.060) Q1+Q4 (0.085)
*KDR*	1.000 0.995	0.115 0.020	0.055 0.025	0.680 0.425	1.000 0.990	1.000 0.985	1.000 0.985	Q1+D (1.000) Q1+D (0.990)
*VEGFA*	0.525 0.305	0.065 0.040	0.055 0.040	0.115 0.090	0.460 0.230	0.320 0.135	0.335 0.135	Q1+D (0.460) Q1+D (0.230)
*VEGFC*	0.785 0.720	0.050 0.045	0.065 0.050	0.325 0.300	0.790 0.665	0.600 0.490	0.625 0.530	Q1+D (0.790) Q1+D (0.665)

We report the powers without (upper) and with (lower) the adjustment of stratification for the single-trait analyses (Q1, Q2, Q4, D), the CMT method for Q1 and D (Q1+D), the CMT method for all the four traits (Q1+Q2+Q4+D), and the OCMT method (OCMT). The last column presents the subset with the highest power among all the subsets with at least two traits and its power (in parentheses).

We used the CMT method with the quadratic statistic to perform the multiple-trait analysis for Q1 and D (Q1+D). For all the Q1 causal genes, the power of the Q1+D analysis was less than or comparable to the power of the univariate test for Q1. The results show that all Q1 causal genes may have small or no pleiotropic effects that are insufficient to compensate for the increase of the critical value from  to . Checking the GAW17 simulation model revealed that this result is reasonable and consistent with the simulation model.

Among all the Q1 causal genes, under the simulation model, only *ELAVL4* was assumed to have pleiotropic effects on Q1 and the latent liability. Moreover, only two rare variants in this gene with MAF = 0.000717 (C1S3181 and C1S3182) contributed to the latent liability. Therefore it could be difficult to detect the association of *ELAVL4* with the latent liability. The other eight causal genes on Q1 were assumed to have no pleiotropic effects. Therefore multiple-trait analysis did not improve the power for Q1 causal genes. However, multiple-trait analysis did elucidate the genetic correlation between the disease status and related quantitative traits and aided in the identification of the pleiotropic genes.

The *p*-value of the OCMT method is the minimum of the *p*-values of all subsets with at least two traits. Multiple-trait analysis was performed for all subsets of Q1, Q2, Q4, and D using the CMT method. The optimally combined multiple traits were selected on the basis of their *p*-values. The power of the OCMT method was comparable with that of the analysis for Q1+Q2+Q4+D. The last column of Table 1 summarizes the subset with the highest power among all the possible subsets with at least two traits and their powers. Most of the genes had the highest power when Q1 and D were combined. This finding shows that the OCMT method provides a way to select the best combination of traits, because the disease status is derived on the basis of multiple traits and Q1 has the biggest effect size.

Table 2 summarizes the power to detect the causal genes on Q2, given a significance level of 0.05. In general, the univariate test for Q2 has the largest power, and the combination of Q2 and D has relatively good performance compared with the other subsets of multiple traits. Table 3 summarizes the power to detect the causal genes on the latent liability. After adjusting for population stratification, all the genes had a power less than 0.3 for both single- and multiple-trait analyses. The power of the analysis for Q1+Q2+Q4+D was less than or comparable to that of the single-trait analysis for D. This result is not surprising, because only *ELAVL4* has a small pleiotropic effect on Q1; the other genes have no effects on Q1, Q2, or Q4.

**Table 2:** Power to detect the causal genes on Q2 at the 0.05 significance level

Gene	Q1	Q2	Q4	D	Q2+D	Q1+Q2 +Q4+D	OCMT	Subset with the highest power (power)
*BHCE*	0.060 0.055	0.445 0.430	0.075 0.060	0.170 0.160	0.340 0.340	0.210 0.190	0.215 0.205	Q2+D (0.340) Q2+D (0.340)
*GCKR*	0.040 0.055	0.415 0.430	0.040 0.020	0.105 0.105	0.380 0.385	0.310 0.330	0.295 0.350	Q1+Q2 (0.455) Q1+Q2 (0.390)
*INSIG1*	0.045 0.035	0.030 0.035	0.055 0.045	0.550 0.045	0.050 0.030	0.070 0.045	0.065 0.035	Q1+Q4+D (0.080) Q1+D (0.065)
*LPL*	0.065 0.070	0.095 0.165	0.055 0.020	0.090 0.045	0.065 0.140	0.125 0.125	0.120 0.130	Q1+D (0.190) Q1+Q2 (0.210)
*PDGFD*	0.020 0.020	0.275 0.300	0.040 0.030	0.105 0.070	0.175 0.225	0.160 0.175	0.155 0.175	Q1+Q2+D (0.190) Q2+D (0.225)
*PLAT*	0.025 0.025	0.050 0.070	0.010 0.010	0.075 0.095	0.060 0.095	0.045 0.045	0.040 0.035	Q2+D (0.060) Q2+D (0.095)
*RARB*	0.225 0.050	0.105 0.070	0.095 0.040	0.075 0.060	0.075 0.055	0.145 0.065	0.135 0.070	Q1+Q2 (0.160) Q1+D (0.070)
*SIRT1*	0.050 0.065	0.605 0.555	0.060 0.050	0.090 0.090	0.530 0.450	0.445 0.410	0.480 0.430	Q1+Q2 (0.545) Q1+Q2 (0.515)
*SREBF1*	0.085 0.035	0.515 0.540	0.055 0.035	0.115 0.100	0.420 0.500	0.410 0.385	0.415 0.395	Q1+Q2 (0.520) Q2+D (0.500)
*VLDLR*	0.025 0.025	0.140 0.230	0.030 0.015	0.090 0.070	0.145 0.210	0.095 0.125	0.100 0.095	Q2+D (0.145) Q2+D (0.210)
*VNN1*	0.465 0.045	0.210 0.050	0.065 0.065	0.115 0.045	0.140 0.050	0.295 0.035	0.285 0.030	Q1+Q2 (0.360) Q1+Q2 (0.065)
*VNN3*	0.035 0.025	0.460 0.380	0.055 0.035	0.070 0.055	0.365 0.275	0.260 0.205	0.280 0.195	Q2+Q4 (0.040) Q2+Q4 (0.280)
*VWF*	0.030 0.025	0.245 0.205	0.050 0.035	0.140 0.075	0.205 0.170	0.115 0.110	0.110 0.120	Q2+D (0.205) Q2+D (0.170)

We report the powers without (upper) and with (lower) the adjustment of stratification for the single-trait analyses (Q1, Q2, Q4, D), the CMT method for Q2 and D (Q2+D), the CMT method for all the four traits (Q1+Q2+Q4+D), and the OCMT method (OCMT). The last column presents the subset with the highest power among all the subsets with at least two traits and its power (in parentheses).

**Table 3:** Power to detect the causal genes on the latent liability at the 0.05 significance level

Gene	Q1	Q2	Q4	D	Q1+Q2 +Q4+D	OCMT	Subset with the highest power (power)
*AKT3*	0.025 0.070	0.045 0.055	0.030 0.020	0.020 0.020	0.040 0.025	0.030 0.030	Q4+D (0.040) Q1+D (0.045)
*BCL2L11*	0.030 0.030	0.050 0.075	0.030 0.020	0.065 0.060	0.055 0.065	0.055 0.070	Q1+Q2+D (0.080) Q2+D (0.095)
*ELAVL4*	0.520 0.045	0.050 0.035	0.060 0.045	0.030 0.045	0.390 0.040	0.380 0.045	Q1 +D (0.465) Q1+Q3 (0.055)
*HSP90AA1*	0.065 0.010	0.050 0.055	0.080 0.040	0.075 0.030	0.095 0.035	0.095 0.045	Q1+Q2+D (0.125) Q1+Q2+D (0.055)
*NRAS*	0.015 0.020	0.035 0.035	0.035 0.010	0.095 0.095	0.010 0.015	0.005 0.010	Q4+D (0.045) Q4+D (0.045)
*PIK3C2B*	0.030 0.055	0.085 0.105	0.025 0.020	0.200 0.145	0.125 0.100	0.115 0.105	Q1+D (0.190) Q1+D (0.145)
*PIK3C3*	0.990 0.280	0.065 0.025	0.045 0.020	0.540 0.200	0.910 0.180	0.915 0.175	Q1+Q4 (0.970) Q1+Q4+D (0.190)
*PRKCA*	0.480 0.025	0.045 0.045	0.045 0.010	0.395 0.115	0.310 0.080	0.355 0.080	Q1+D (0.425) Q2+D (0.100)
*PRKCB1*	0.025 0.045	0.055 0.060	0.035 0.035	0.035 0.025	0.030 0.045	0.045 0.040	Q2+Q4+D (0.045) Q1+Q2+Q4+D (0.045)
*PTK2*	0.660 0.095	0.075 0.025	0.020 0.015	0.160 0.050	0.405 0.035	0.400 0.060	Q1+D (0.530) Q1+D (0.105)
*PTK2B*	1.000 0.190	0.235 0.020	0.040 0.055	0.525 0.090	0.990 0.135	0.995 0.130	Q1+D (0.995) Q1+Q4+D (0.155)
*RRAS*	0.025 0.040	0.045 0.035	0.045 0.025	0.090 0.085	0.050 0.040	0.045 0.045	Q2+Q4 (0.040) Q2+Q4 (0.280)
*SHC1*	0.280 0.035	0.020 0.030	0.065 0.045	0.105 0.045	0.150 0.015	0.155 0.015	Q1+Q2+D (0.065) Q2+D (0.090)
*SOS2*	0.840 0.105	0.065 0.065	0.030 0.015	0.180 0.090	0.580 0.080	0.610 0.085	Q1+D (0.745) Q1+Q2+D (0.105)

We report the powers without (upper) and with (lower) the adjustment of stratification for the single-trait analyses (Q1, Q2, Q4, D), the CMT method for all the four traits (Q1+Q2+Q4+D), and the OCMT method (OCMT). The last column presents the subset with the highest power among all the subsets with at least two traits and its power (in parentheses).

### False-positive rates of single- and multiple-trait analyses

On the basis of the results adjusted for population stratification, we calculated the false-positive rates of single- and multiple-trait analyses. Genes with a power greater than or equal to 0.3 were considered the associated findings. Luedtke et al. [12] reported that 695 genes were spuriously associated with the disease status. Excluding these 695 genes, we identified 10 causal genes (4 on Q1, 6 on Q2, and 3 on D) and 77 false-positive genes (62 on Q1, 14 on Q2, 0 on Q4, and 6 on D) in the single-trait analysis for Q1, Q2, Q4, and D. The false-positive rate of the single-trait analysis was equal to 0.031. The multiple-trait analysis Q1+Q2+Q4+D detected six causal genes and 58 false-positive genes. The false-positive rate of the Q1+Q2+Q4+D analysis was equal to 0.023. This result shows that, compared with the single-trait analysis, the CMT method combining multiple traits does not increase the false-positive rate.

## Discussion

In some genetic association studies, multiple correlated traits are available that can be used to identify genes responsible for multiple traits. In single-trait analysis, some common associated genes may be found across different traits. These overlapping associations may be caused by pleiotropic genes and/or the correlation structure among traits. Multiple-trait analysis has the potential to improve the power to detect pleiotropic genes. The multiple-trait analysis proposed here did not suffer a significant loss of power, even though true models, such as the GAW17 simulated model, had small or no pleiotropic effects. The proposed method considers the correlation matrix, thereby ensuring that the false-positive rate is not inflated by the correlation among multiple traits.

In next-generation sequencing data sets, huge numbers of rare variants are genotyped across the whole genome. Because of the small number of observations for each rare variant, statistical tests for common variants are inefficient at identifying the associations. Hence the proposed method performs a gene-level test using the weighted sum to test the joint effect of multiple variants within a gene. The weighted sum is a feasible choice for the gene-based score [8], but it is not the only choice; other methods are available for pooling multiple rare variants [4].

From Tables 1 and 3, it can be seen that the correction for population stratification has a large effect on the power to detect some associations with Q1, D, and Q1+Q2+Q4+D. This phenomenon also was observed from the quantile-quantile plots in replicate 1 (data not shown). Although we did not consider the population structure in the simulation model, the principal component scores may influence the phenotypes, because the population structures would be similar to those of the true causal genes in this data set [13]. We conclude that the rare variant associations unadjusted for population stratifications should be interpreted with caution.

## Conclusions

With the advent of next-generation sequencing technologies, the identification of rare variants has become realistic. Statistical methods for common variants are not applicable in rare variant analysis because of the small number of observations and the huge number of rare variants across the whole genome. Thus efficient statistical methods are needed. When multiple related traits are available, it is expected that multiple-trait analysis has the improved power to detect pleiotropic genes. We proposed the CMT and OCMT methods to examine the joint effect of multiple variants on multiple traits. The proposed method uses the quadratic or linear combination of univariate test statistics and thus considers the correlation structure among multiple correlated traits. The CMT and OCMT methods were applied to the GAW17 mini-exome data. The results show that the method is suitable for multiple trait analysis.

## Competing interests

The authors declare that they have no competing interests.

## Authors’ contributions

JZ conceived of the study, participated in its design and wrote the paper. AT participated in the design of the study and made the major edits. Both authors read and approved the final manuscript.

## References

[B1] Hirschnorm JN Daly MJ Genome-wide association studies for common disease and complex traits Nat Rev Genet 2005 6 95 108 15716906 10.1038/nrg1521

[B2] Iyengar SK Elston RC The genetic basis of complex traits: rare variants or “common gene, common disease”? Meth Mol Biol 2007 376 71 84 10.1007/978-1-59745-389-9_6 17984539

[B3] Cohen JC Boerwinkle E Mosley THJ Hobbs HH Multiple rare alleles contribute to low plasma levels of HDL cholesterol Science 2004 305 869 872 10.1126/science.1099870 15297675

[B4] Dering C Pugh E Ziegler A Statistical analysis of rare variants: An overview of collapsing methods Genet Epidemiol 2011 X X X 10.1002/gepi.20643 PMC3277891 22128052

[B5] Jiang C Zheng ZB Multiple trait analysis of genetic mapping for quantitative trait loci Genetics 1995 140 1111 1127 7672582 10.1093/genetics/140.3.1111 PMC1206666

[B6] Liu XG Liu YJ Liu J Pei Y Xiong DH Shen H Deng HY Papasian CJ Drees BM Hamilton JJ A bivariate whole genome linkage study identified genomic regions influencing both BMD and bone structure J Bone Mineral Res 2008 23 1806 1814 10.1359/jbmr.080614 PMC2685488 18597637

[B7] Luo L Peng G Zhu Y Dong H Amons CI Xiong M Genome-wide gene and pathway analysis Eur J Hum Genet 2010 18 1045 1053 10.1038/ejhg.2010.62 20442747 PMC2924916

[B8] Madsen BE Browning SR A groupwise association test for rare mutations using a weighted sum statistic PLoS Genet 2009 5 e1000384 10.1371/journal.pgen.1000384 19214210 PMC2633048

[B9] Bickel PJ Doksum KA Mathematical Statistics: Basic Ideas and Selected Topics 2001 2nd Upper Saddle River, NJ, Prentice Hall, v. 1 506 508

[B10] Almasy LA Dyer TD Peralta JM Kent JW Jr. Charlesworth JC Curran JE Blangero J Genetic Analysis Workshop 17 mini-exome simulation BMC Proc 2011 5 suppl 9 S2 22373155 10.1186/1753-6561-5-S9-S2 PMC3287854

[B11] Price AL Patterson NJ Plenge RM Weinblatt ME Shadick NA Reich D Principal component analysis corrects for stratification in genome-wide association studies Nat Genet 2006 38 904 909 10.1038/ng1847 16862161

[B12] Luedtke A Powers S Petersen A Sitarik A Bekmetjev A Tintle NL Evaluating methods for the analysis of rare variants in sequence data BMC Proc 2011 5 suppl 9 S119 10.1186/1753-6561-5-S9-S119 PMC3287843 22373354

[B13] Qin H Elston R Zhu X Interrogating population structure and its impact on association tests BMC Proc 2011 5 suppl 9 S25 22373290 10.1186/1753-6561-5-S9-S25 PMC3287860

